# MiR-27a-3p/Hoxa10 Axis Regulates Angiotensin II-Induced Cardiomyocyte Hypertrophy by Targeting Kv4.3 Expression

**DOI:** 10.3389/fphar.2021.680349

**Published:** 2021-06-24

**Authors:** Xuefeng Cao, Zheng Zhang, Yu Wang, Weichao Shan, Ruiting Wang, Shufang Mao, Shi Ding, Chong Pang, Baoqun Li, Jian Zhou, Xiaoyan Guo, Na Guo, Cui Li, Jing Liang, Wenya Ma, Yu Liu, Liang Zhao

**Affiliations:** ^1^ Department of Basic Medicine, Chengde Medical College, Chengde, China; ^2^ Department of Anesthesiology, Affiliated Hospital of Chengde Medical College, Chengde, China; ^3^ The First Affiliated Hospital of Harbin Medical University, Harbin, China; ^4^ Department of Pediatric Orthopedics, Affiliated Hospital of Chengde Medical College, Chengde, China; ^5^ Department of Cardiology, Affiliated Hospital of Chengde Medical College, Chengde, China; ^6^ Academic Affairs, Chengde Medical College, Chengde, China; ^7^ State Key Laboratory of Experimental Hematology, National Clinical Research Center for Blood Diseases, Institute of Hematology and Blood Diseases Hospital, Chinese Academy of Medical Sciences and Peking Union Medical College, Tianjin, China; ^8^ The Second Affiliated Hospital of Harbin Medical University, Harbin, China; ^9^ The Forth Affiliated Hospital of Harbin Medical University, Harbin, China

**Keywords:** cardiac hypertrophy, electrical remodeling, HOXA10, miRNA-27a-3p, Ang II

## Abstract

Cardiac hypertrophy is a common pathological process of various cardiovascular diseases, which is often accompanied with structural and electrical remodeling, and can even lead to sudden cardiac death. However, its molecular mechanism still remains largely unknown. Here, we induced cardiomyocyte hypertrophy by angiotensin II (Ang II), and found that miR-27a-3p and hypertrophy-related genes were up-regulated. Further studies showed that miR-27a-3p-inhibitor can alleviate myocardial hypertrophy and electrical remodeling. Moreover, luciferase assay confirmed that miR-27a-3p could regulate the expression of downstream *Hoxa10* at the transcriptional level by targeting at its 3′UTR. At the same time, the protein expression of Hoxa10 was significantly reduced in Ang II-treated cardiomyocytes. Furthermore, overexpression of *Hoxa10* can reverse myocardial hypertrophy and electrical remodeling induced by Ang II in cardiomyocytes. Finally, we found that Hoxa10 positively regulated the expression of potassium channel protein Kv4.3 which was down-regulated in hypertrophic cardiomyocytes. Taken together, our results revealed miR-27a-3p/Hoxa10/Kv4.3 axis as a new mechanism of Ang II-induced cardiomyocyte hypertrophy, which provided a new target for clinical prevention and treatment of cardiac hypertrophy and heart failure.

## Introduction

Myocardial hypertrophy is a common pathological process of cardiovascular diseases such as hypertension. Long-term myocardial hypertrophy leads to cardiac function decompensation, aggravates cardiac electrical remodeling, greatly increases the susceptibility to arrhythmia (Thompson, 2009; Dey et al., 2018), and even leads to heart failure and sudden cardiac death (SCD) (Coyne et al., 2001). Epidemiological investigation has confirmed that the incidence of arrhythmia, heart failure and sudden cardiac death in patients with myocardial hypertrophy is much higher than normal cohorts (Berger et al., 2002; John et al., 2012). However, current medication or surgery therapy cannot effectively improve the long-term survival rate of patients with myocardial hypertrophy. Therefore, the pathogenesis of cardiac hypertrophic has increasingly become a focus and difficulty in the field of cardiovascular diseases, and it is also of great importance to reveal the mechanism of myocardial remodeling and new targets for clinical treatment of cardiac hypertrophy (Gatzoulis et al., 2012). Although more and more researchers are interested in the study of myocardial hypertrophy and electrical remodeling, the molecular mechanisms of myocardial hypertrophy and electrical remodeling still remain largely unknown (Bacharova, 2019).

Micro-RNA (microRNA, miRNA) is a kind of non-coding RNA with approximately 22 nucleotides, which can inhibit the translation of target gene mRNAs by incomplete complementary binding to the 3′UTR region of target gene mRNAs, and then degrade the expression of mRNA (Yang et al., 2007). A large number of studies have shown that miRNAs play an important role in the occurrence and development of heart diseases, especially in cardiomyopathy such as myocardial hypertrophy and arrhythmia (Wang et al., 2008; Samidurai et al., 2018). For example, muscle-specific microRNA miR-1 has been shown to promote the development of myocardial ischemia and apoptosis in mice. MiRNAs such as miR-101 was linked to cardiac fibrosis and fibroblast transdifferentiation via targeting c-Jun (Pan et al., 2012). Although several studies have revealed that miR-27a-3p is associated with several cardiovascular diseases, its roles in myocardial hypertrophy and electrical remodeling still needs to be further clarified (Chen et al., 2012).

In this study, we observed that the expression of miR-27a-3p was up-regulated in cardiac hypertrophy. MiR-27a-3p-inhibitor could inhibit myocardial hypertrophy and electrical remodeling induced by Ang II. Further studies showed that miR-27a-3p binded to 3′UTR of *Hoxa10* which was down-regulated in cardiac hypertrophy. The overexpression of *H*oxa10 could reverse cardiac hypertrophy and electrical remodeling by targeting Kv4.3 protein. Therefore, our results revealed for the first time that the miR-27a-3p/Hoxa10/Kv4.3 axis is involved in the pathogenesis of myocardial hypertrophy and electrical remodeling, and these results provided a new therapeutic strategy for myocardial hypertrophy and electrical remodeling clinically.

## Materials and Methods

### Animals and Ethical Approval

Sprague-Dawley rats (1–3 days, SPF grade, Certification No. 11400700368598) were obtained from the Beijing Huafukang Biological Technology Co. Ltd. (Beijing, China). The experimental protocols were approved by the Ethics Committee of the Ethics of Animal Experiments of Chengde Medical College (Approval ID: CDMULAC-20191031-009). The animal experiments were approved by the Research Ethics Committee of Chengde Medical College, and were in accordance with the Guide for the Care and Use of Laboratory Animals (NIH Publication No. 85-23, revised 1996).

### Primary Culture and Treatment of Neonatal Rat Cardiomyocytes

Cardiomyocytes were obtained from 1 to 3-day-old neonatal rats as previously described, with a few minor modifications (Cai et al., 2018). Briefly, after being digested in 0.25% trypsin solution, myocytes were isolated by selective adhesion of myocytes at a 2 h pre-plating interval. Cardiomyocytes were cultured in DMEM supplemented with 1% penicillin and streptomycin, 10% fetal bovine serum and 0.1 mM 5-bromo-2-deoxyuridine in a 37°C incubator with 5% CO_2_. MiR-27a-3p-mimics (5′-UUC​ACA​GUG​GCU​AAG​UUC​CGC-3′), miR-27a-3p-inhibitor (5′-GCG​GAA​CUU​AGC​CAC​UGU​GAA-3′), *Hoxa10*-overexpress and corresponding NC were synthesized by Shanghai Sangon Biotechnology, and transiently transfected with liposome3000 transfection reagent (Invitrogen, Carlsbad, CA, United States) and serum-free medium. Eight hours after transfection, a new serum-free medium with or without Ang II (10 μM) was used instead. After 48 h of culture, the cells were collected for protein/total RNA extraction (Ma et al., 2018).

### Western Blot Analysis

After treatments, the cardiomyocytes were washed with PBS for three times, then the protein lysate was added and stored at −80°C. Place cells on the ice to dissolve, scrape off the cells with a protein scraper and centrifuge at 13,500 rpm/min × 15 min, 4°C. Collect the supernatant protein solution. Determination of protein concentration by BCA method, proteins were separated by SDS-PAGE and transferred to a PVDF membrane. After blocking with 5% fat-free milk at room temperature for 120 min, the membranes were incubated overnight with the corresponding primary antibody at 4°C, including anti-Kv4.3 (1:1,000, CST), anti-Hoxa10 (1:500, Sigma), anti-ANP (1:1,000, SANTA), anti-BNP (1:1,000, SANTA), anti-MHC (1:1,000, SANTA) and anti-GAPDH (1:10,000, ABclonal). After being washed with TBST by three times, each time 10 min, the membranes were incubated with secondary antibodies (1:8,000, ABclonal) for 2 h at room temperature and washed with TBST again. The proteins were scanned using Odyssey version 1.2 (LI-COR Biosciences, Lincoln, NE, United States) and Image Studio software.

### Real-Time Quantitative RT-PCR

Total RNA samples from different groups of cardiomyocytes were extracted and treated with DNA enzyme 1, and then routine quantitative RT-PCR (qRT-PCR) was used for detection. Real-time quantitative RT-PCR was determined by Taqman probe method (ABI 7500 fast). In the determination of mRNA, GAPDH was used as the internal reference, and U6 was used as the internal reference for microRNAs. Compared with the control group, the related expressions of microRNAs and mRNAs under different treatments were calculated.

### Immunofluorescent Chemical Staining

Cardiomyocytes in different treatment groups were washed with PBS for three times, and 15 min was fixed with 4% paraformaldehyde at 37°C. Get rid of paraformaldehyde, wash the cells with PBS, then add a mixed solution (0.4% Triton + 10 mg BSA +1 ml PBS) and place 90 min at room temperature. After that, goat serum blocked 30 min at 37°C, then α-actinin was added and incubated at 4°C for 48 h. Finally, after incubated with fluorescent second antibody for 1 h, the cells were washed by PBS for three times, and the morphological changes of cells were observed by fluorescence microscope.

### Statistical Analysis

GraphPad Prism software was used for statistical analysis. Values were expressed as the mean ± SEM. Differences between two groups were analyzed using *t*-test. The significance of the difference between groups in review were analyzed by one-way analysis of variance (ANOVA). A value of *p < 0.05, **p < 0.01, ***p < 0.001 and ****p < 0.0001 were considered statistically significant difference for all analyses.

## Results

### Abnormal Expression of MiR-27a-3p During Cardiomyocyte Hypertrophy Induced by Ang II

**FIGURE 1:**
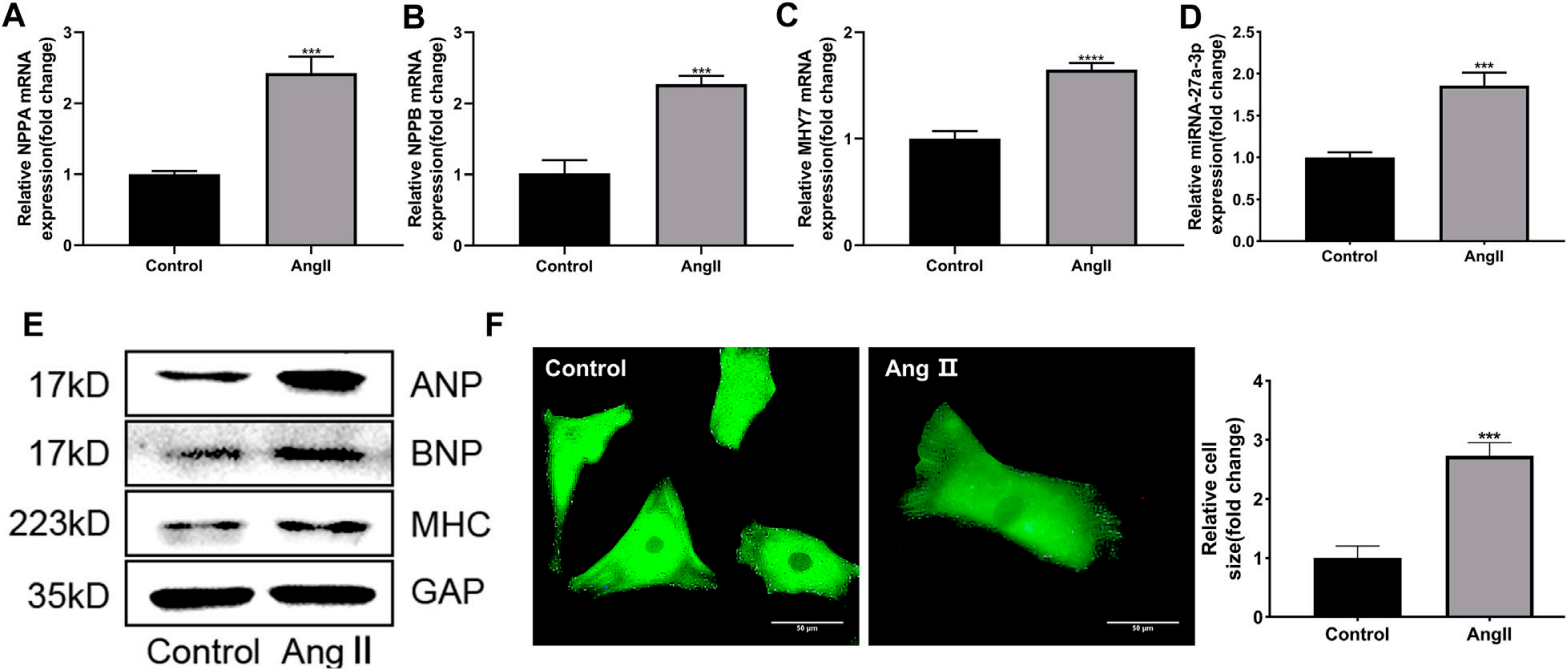
Ang II induced cardiac hypertrophy in neonatal rat cardiomyocytes. **(A–C)** The expressions of cardiac hypertrophy related genes *NPPA*, *NPPB* and *MHY7* were increased after Ang II treatment. **(D)** The expression of miR-27a-3p increased after Ang II treatment. **(E)** The protein expressions of cardiac hypertrophy related genes ANP, BNP and β-MHC were increased after Ang II treatment. **(F)** Morphological changes after Ang II treatment in neonatal rat cardiomyocytes. Scale bar, 50 μm. All the values are the mean of three independent experiments (*n* = 3). ****p < 0.0001, ***p < 0.001 vs. control.

Firstly, the cardiomyocytes from rats were extracted and exposed to Ang II (10 μM) for 48 h to established cardiac hypertrophy cell model *in vitro*. Then the expressions of myocardial hypertrophy-related genes and miRNAs in cardiomyocytes were detected. Results showed that the expressions of myocardial hypertrophy-related genes and proteins including ANP, BNP and β-MHC were markedly up-regulated in Ang II-treated cardiomyocytes compared with control group (Figures 1A,B,C,E). Immunofluorescence results showed that the area of cardiomyocytes in Ang II treatment group was significantly larger than the control group (Figure 1F), indicating that Ang II induced cardiac hypertrophy cell model *in vitro* was successfully established. At the same time, we also found that the expression of miR-27a-3p was up-regulated in cardiomyocytes after Ang II treatment compared with control group (Figure 1D). This result suggested that miR-27a-3p might be involved in myocardial hypertrophy induced by Ang II.

### MiR-27a-3p-Inhibitor Mitigates Cardiomyocyte Hypertrophy Induced by Ang II

**FIGURE 2:**
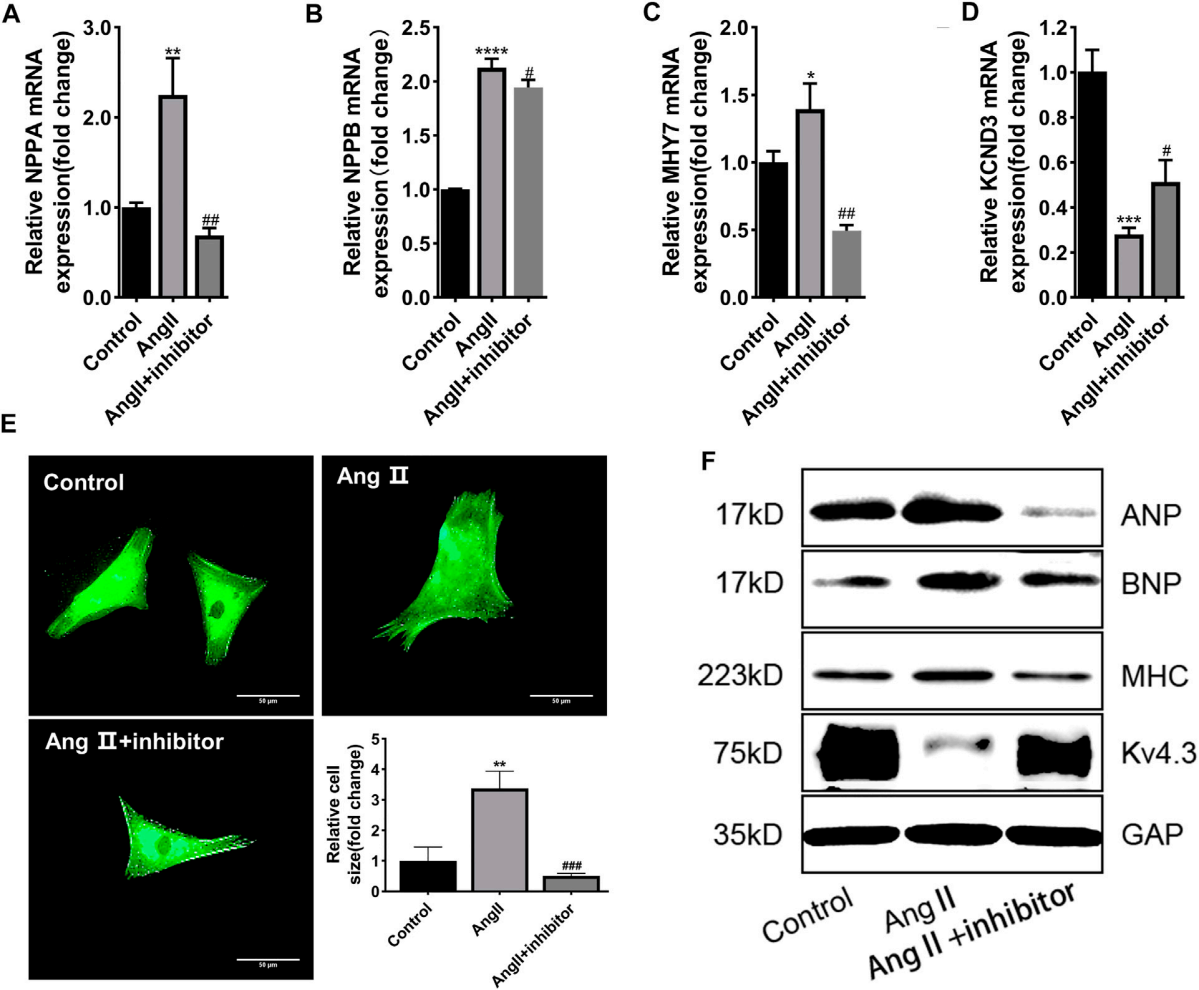
MiR-27a-3p is involved in the process of myocardial hypertrophy induced by Ang II. **(A–C)** The expressions of cardiac hypertrophy related genes *NPPA, NPPB,* and *MHY7* were decreased after Ang II + miR-27a-3p inhibitor treatment compared with the Ang II treatment. **(D)** The expression of *KCND3* decreased after Ang II treatment, while increased in Ang II + miR-27a-3p inhibitor treatment. **(E)** Morphological changes after Ang II and miR-27a-3p inhibitor treatment in neonatal rat cardiomyocytes. Scale bar, 50 μm. **(F)** The protein expressions of cardiac hypertrophy related genes ANP, BNP and β-MHC were decreased, while the expression of Kv4.3 increased after Ang II + miR-27a-3p inhibitor treatment. All the values are the mean of three independent experiments (*n* = 3). ****p < 0.0001, ***p < 0.001, **p < 0.01, *p < 0.05 vs. control. ^###^ *p* < 0.001, ^##^ *p* < 0.01, ^#^ *p* < 0.05 vs. Ang II.

To clarify whether miR-27a-3p was involved in cardiac hypertrophy, miR-27a-3p-inhibitor, a specific antagonist of miR-27a-3p, was used in the following experiments. Four hours after the transfection of miR-27a-3p-inhibitor, the serum-free culture medium was replaced and co-cultured with Ang II (10 μM) for 48 h. PCR results showed that the expressions of *NPPA, NPPB*, and *MHY7* in miR-27a-3p-inhibitor + Ang II group was significantly lower than Ang II group, while *KCND3* was higher than Ang II group (Figures 2A–D), and the protein expressions showed similar tendencies (Figure 2F). The α-actinin staining showed that the area of cardiomyocytes in miR-27a-3p-inhibitor + Ang II group was significantly smaller than Ang II group (Figure 2E). These results indicated that the inhibition of miR-27a-3p mitigated the process of myocardial hypertrophy and structural remodeling induced by Ang II.

### MiR-27a-3p-Mimics Promotes Hypertrophic Growth in Rat Cardiomyocytes

**FIGURE 3:**
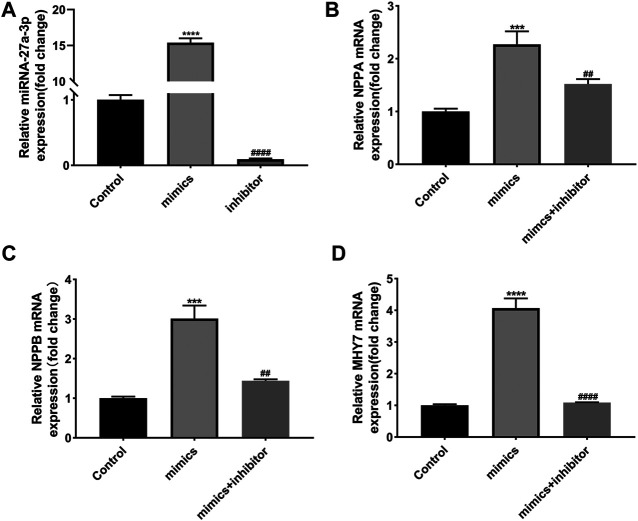
Effects of miR-27a-3p-mimics and inhibitor on cardiomyocytes. **(A)** The expression of miR-27a-3p in miR-27a-3p-mimics and miR-27a-3p-inhibitor treatment group. **(B–D)** The expressions of *NPPA, NPPB,* and *MHY7* in miR-27a-3p-mimics treatment group and miR-27a-3p-mimics + miR-27a-3p-inhibitor co-treatment group. All the values are the mean of three independent experiments (*n* = 3). ****p < 0.0001, ***p < 0.001 vs. control, ^###^ *p* < 0.0001, ^##^ *p* < 0.01 vs. mimics.

In addition, we detected the effects of miR-27a-3p-mimics and inhibitor on cultured rat ventricular cardiomyocytes. PCR results showed that the expression of miR-27a-3p in miR-27a-3p-mimics group was significantly increased compared with control group, while the expression of miR-27a-3p in miR-27a-3p-inhibitor group was significantly decreased compared with miR-27a-3p-mimics group and the control group (Figure 3A). Furthermore, the expressions of *NPPA*, *NPPB*, and *MHY7* were significantly up-regulated after miR-27a-3p-mimics treatment, which were effectively alleviated by miR-27a-3p-mimics and miR-27a-3p-inhibitor co-treatment (Figures 3B–D). These results suggested that the overexpression of miR-27a-3p owned the potential to promote cardiomyocyte hypertrophy.

### MiR-27a-3p Regulates the Expression of Kv4.3 in Rat Ventricular Cardiomyocytes

**FIGURE 4:**
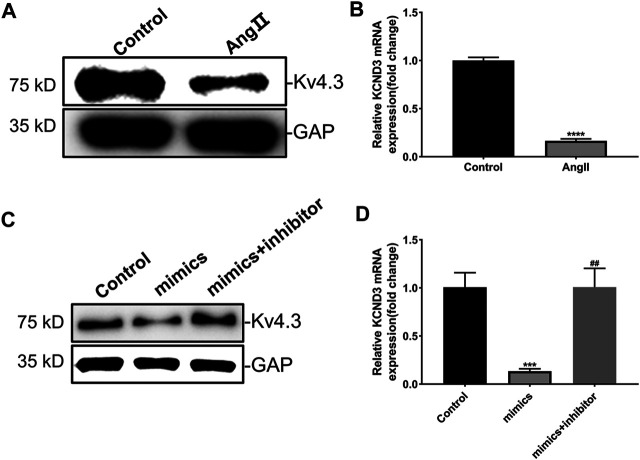
MiR-27a-3p negatively regulates the expression of Kv4.3 in rat ventricular cardiomyocytes. **(A–B)** The expressions of Kv4.3 channel protein and KCND3 gene were down-regulated in Ang II-induced cardiomyocyte hypertrophy. **(C–D)** MiR-27a-3p-inhibitor can abrogate the changes in the expression of KCND3/Kv4.3 induced by miR-27a-3p-mimics. All the values are the mean of three independent experiments (*n* = 3). ****p < 0.0001, ***p < 0.001 vs. control, ^##^ *p* < 0.01 vs. mimics.

The results above proved that miR-27-3p was involved in hypertrophic growth of cardiomyocytes, but its mechanism still needs to be elucidated. Recent study revealed that potassium channel gene *KCND3* acts as an important mediator in cardiomyocyte hypertrophy (Wang et al., 2019). Consistently, we found that the expressions of Kv4.3 protein and KCND3 gene were down-regulated in Ang II-induced cardiomyocyte hypertrophy (Figures 4A,B). Furthermore, miR-27-3p-mimics transfection induced a reduction expression of Kv4.3 channel protein in cardiomyocytes. On the contrary, miR-27-3p-mimics and miR-27-3p-inhibitor co-treatment attenuated the reduction of Kv4.3 protein expression in hypertrophic cardiomyocytes, and PCR results showed the similar tendencies (Figures 4C,D). These results indicated that miR-27-3p regulated cardiomyocyte hypertrophy by affecting the expression of Kv4.3 channel protein. However, no binding sites of miR-27-3p in the 3′UTR region of KCND3 gene were found.

### MiR-27a-3p Negatively Regulates Hoxa10 Expression by Directly Binding to 3ʹUTR of *Hoxa10*

**FIGURE 5:**
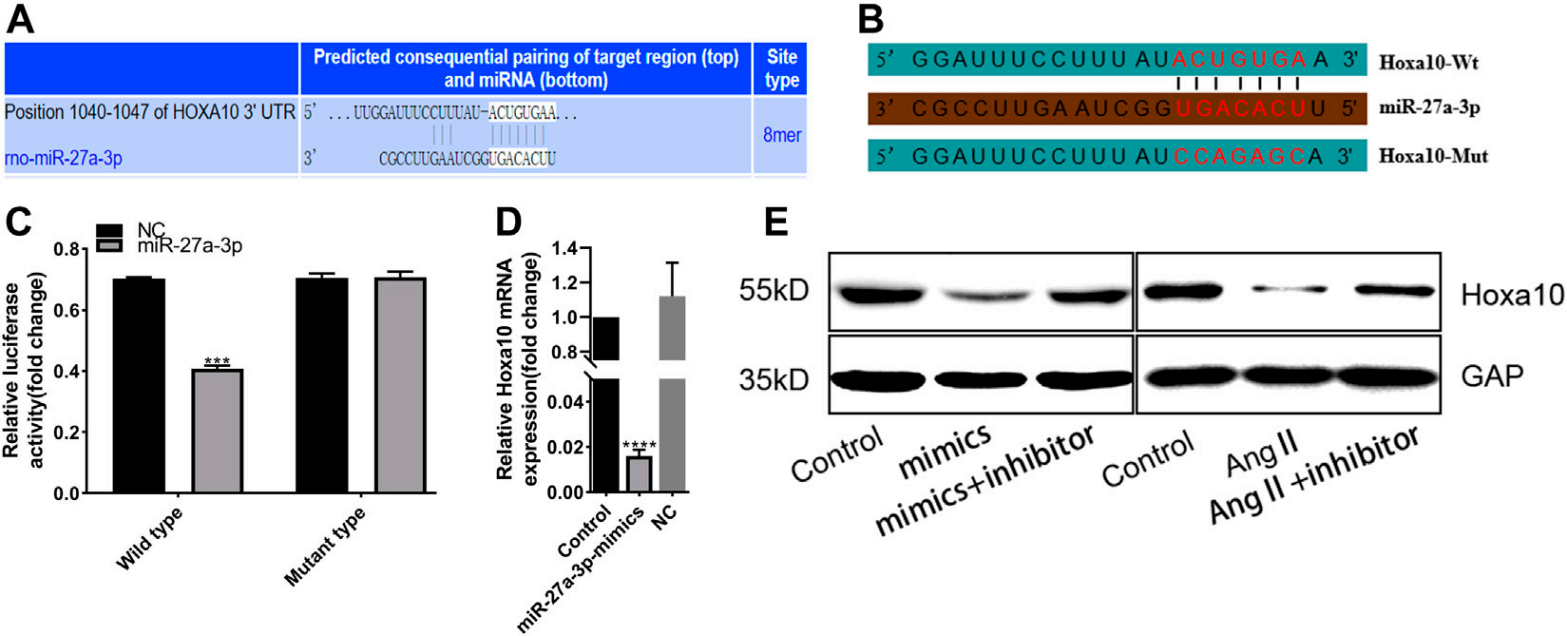
MiR-27a-3p can negatively regulate the expression of Hoxa10. **(A)** The binding sequence of miR-27a-3p and 3′UTR region of *Hoxa10* was predicted by TargetScan. **(B)** Wt 3′UTR and Mut 3′UTR of *Hxoa10* were constructed. **(C)** Luciferase assay confirmed that miR-27a-3p had transcriptional regulation effect on *Hoxa10*. **(D)** The expression of Hoxa10 was decreased in miR-27a-3p-mimics treatment group compared with the control and NC group. **(E)** Western blot was applied to detect the protein expression of Hoxa10 after treatment with miR-27a-3p-mimics and miR-27a-3p-inhibitor, as well as the Ang II and Ang II + miR-27a-3p inhibitor treatment. All the values are the mean of three independent experiments (*n* = 3). ****p < 0.0001, ***p < 0.001 vs. NC.

Next, we further searched the downstream targets of miR-27a-3p in the pathogenesis of cardiac hypertrophy and electrical remodeling. The TargetScan prediction showed that there was a binding sequence between miR-27a-3p and *Hoxa10*-mRNA 3′UTR region (Figure 5A). As shown in Figure 5B, we designed wild and mutant types of plasmids of *Hoxa10* 3′UTR region and transfected into HEK293T cells. Luciferase assay showed that the fluorescence intensity of miR-27a-3p group was significantly lower than the NC group in wild type, but there was no change in mutant type (Figure 5C). In addition, compared with NC group, the mRNA expression of *Hoxa10* was significantly inhibited by miR-27a-3p-mimics in cardiomyocytes (Figure 5D). These results confirmed that there was a binding between miR-27a-3p and *Hoxa10*-mRNA 3′UTR region. Furthermore, miR-27a-3p-mimics significantly inhibited the expression of Hoxa10, while this process could be alleviated by miR-27a-3p-mimics and miR-27a-3p-inhibitor co-treatment. Besides, miR-27a-3p-inhibitor can also alleviate the decreased expression of Hoxa10 induced by Ang II treatment (Figure 5E). These results suggested that miR-27a-3p could negatively regulate the expression of Hoxa10.

### Hoxa10 Reversed the Process of Myocardial Hypertrophy and Kv4.3 Reduction Induced by Ang II

**FIGURE 6:**
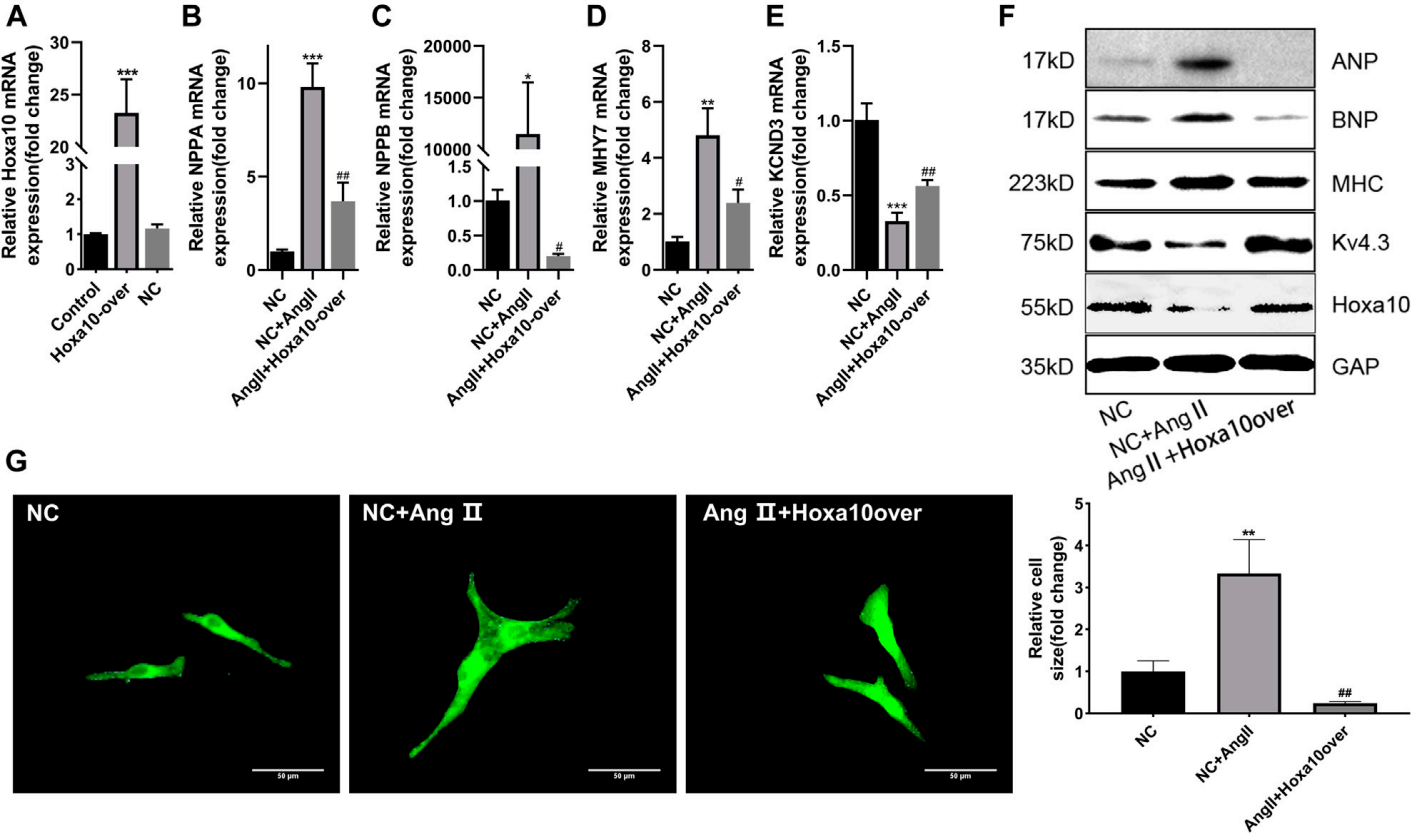
Hoxa10 reverses the process of myocardial hypertrophy and Kv4.3 reduction induced by Ang II. **(A)** The expression of Hoxa10 in Hoxa10-overexpress group was significantly increased compared with the NC group. **(B–D)** The expression of cardiac hypertrophy related genes *NPPA, NPPB,* and *MHY7* in NC + Ang II group was significantly up-regulated compared with the NC group, while down-regulated after treatment with Hoxa10 overexpression. **(E)** The expression of KCND3 in NC + Ang II group was significantly down-regulated compared with the NC group, while up-regulated after Hoxa10 overexpression treatment. **(F)** The protein expressions of ANP, BNP, β-MHC, and Kv4.3 showed similar tendencies as PCR results. The protein expression of Hoxa10 was down-regulated in NC + Ang II group, while up-regulated in Hoxa10-overexpress treatment group **(G)** Morphological changes after the overexpression of *Hoxa10* showed that the area of cardiomyocytes in Ang II + Hoxa10-overexpress group was significantly smaller than that in NC + Ang II group. Scale bar, 50 μm. All the values are the mean of three independent experiments (*n* = 3). ***p < 0.001, **p < 0.01, *p < 0.05 vs. NC, ^###^ *p* < 0.001, ^##^ *p* < 0.01, ^#^ *p* < 0.05 vs. NC + Ang II.

In order to confirm whether Hoxa10 was involved in myocardial hypertrophy process. The cardiomyocytes were cultured with Hoxa10-overexpress for 8 h, then the serum-free medium was replaced, and the cells were co-cultured with Ang II for 48 h. PCR results showed that the mRNA expression of *Hoxa10* in Hoxa10-overexpress group was significantly higher than that in NC group (Figure 6A). Compared with NC group, the expression of cardiac hypertrophy related genes including *NPPA, NPPB,* and *MHY7* in NC + Ang II group was significantly up-regulated, while KCND3 was down-regulated (Figures 6B–E). As shown in Figure 6F, the protein expressions showed similar tendencies. Moreover, the protein expression of Hoxa10 was also down-regulated synchronously in NC + Ang II group. While this process could be inhibited by Hoxa10-overexpress (Figure 6B–F). The α-actinin staining showed that the area of cardiomyocytes in Ang II + Hoxa10-overexpress group was significantly smaller in NC + Ang II group, indicating that Hoxa10-overexpress reversed the process of myocardial hypertrophy induced by Ang II (Figure 6G). To sum up, these results suggested that by targeting the miR-27a-3p/Hoxa10/Kv4.3 axis could effectively inhibit the process of myocardial hypertrophy and cardiomyocyte hypertrophy, which provided us a new therapeutic strategy for myocardial hypertrophy and electrical remodeling.

## Discussion

This study confirmed that Ang II can lead to cardiomyocyte hypertrophy and electrophysiological remodeling. This process is related to the up-regulation of miR-27a-3p and the down-regulation of Hoxa10. We found that miR-27a-3p-inhibitor could mitigate the abnormal mRNA and protein expressions of *NPPA/NPPB/MHY7/KNCD3* and ANP/BNP/β-MHC/Kv4.3 induced by Ang II. Further study confirmed that miR-27a-3p-mimics could inhibit the expression of Hoxa10. Overexpression of *Hoxa10* could reverse the myocardial hypertrophy process induced by Ang II. Collectively, miR-27a-3p and Hoxa10 are involved in cardiac hypertrophy and electrical remodeling induced by Ang II, and the reversal of this process was closely related to targeting miR-27a-3p, Hoxa10 and Kv4.3.

Recent studies have shown that HOX is involved in the regulation of embryonic development, cell differentiation, cell cycle, apoptosis and other biological processes (Sanchez-Herrero, 2013). A large number of reports have pointed out that Hox is related to the occurrence and development of a variety of tumors, such as breast cancer, colorectal cancer, gastric cancer, lung cancer, ovarian cancer and so on (Grier et al., 2005). Although some studies have found that Hoxa10 was closely associated with the occurrence and development of cardiovascular diseases, there are no reports about its implication in myocardial hypertrophy and electrical remodeling (Ng et al., 2016; Souilhol et al., 2020). Our research demonstrated that the overexpression of *H*oxa10 can reverse the expressions of hypertrophy-related genes and electrical remodeling, which is the first time to report the correlation between Hoxa10 and myocardial hypertrophy at home and abroad.

Previous studies have shown that miRNA plays an important role in the occurrence and development of heart disease, especially in cardiomyopathy such as myocardial hypertrophy and arrhythmia (Austin et al., 2019; Colpaert and Calore, 2019). Some abnormally expressed miRNA can regulate the target genes through incomplete complementary binding with the 3′UTR of the target mRNA. Previous studies have shown that the application of arsenic trioxide injection in the treatment of acute promyelocytic leukemia is often accompanied by cardiac side effects, which seriously limits the clinical application of arsenic trioxide (Bao et al., 2019). miR-1 and miR-133 are important targets for cardiac side effects such as arrhythmia and sudden cardiac death caused by arsenic trioxide (Shan et al., 2013). In addition, the expression of miR-133 and miR-590 in the atrium of smokers with atrial fibrillation and nicotine-induced atrial fibrillation was down-regulated, and the protein expressions of target genes TGF-β1 and TGF-β RII was up-regulated, resulting in increased collagen production of atrial fibroblasts, which in turn induced atrial fibrosis and atrial fibrillation (Shan et al., 2009). These reports confirmed that miRNA is closely related to the pathophysiological process of cardiovascular system. In recent years, the role of miR-27a-3p in cardiovascular diseases has become more and more prominent. Researches showed that miR-27a-3p can not only reduce cardiomyocyte injury induced by hypoxia/reoxygenation and lipopolysaccharide (Lozano-Velasco et al., 2015; Zhang et al., 2019), but also promote vascular maturation and angiogenesis by inhibiting SEMA6A and SEMA6D (Zhou et al., 2011; Urbich et al., 2012; Demolli et al., 2017). In addition, it has been reported that miR-27a-3p is closely related to ventricular formation, obesity and cardiac effects in mice (Chinchilla et al., 2011; Sassoon et al., 2016). Our research confirmed that miR-27a-3p could bind to the 3′UTR region of *Hoxa10*, regulate its transcription and inhibit the protein expression, which further led to cardiomyocyte hypertrophy and electrical remodeling. We firstly demonstrated that miR-27a-3p/Hoxa10 axis was involved in myocardial hypertrophy and electrical remodeling.

The limitations of our study are as followings: first of all, we only discussed myocardial hypertrophy and electrical remodeling from the miR-27a-3p/Hoxa10/Kv4.3 axis, but did not further study myocardial regeneration and myocardial fibrosis. Secondly, we only detected the change of miR-27a-3p/Hoxa10/Kv4.3 *in vitro*, but did not verify the changes *in vivo*. Due to the reason of time, we did not further carry out this study.

In summary, our study suggested that miR-27a-3p/Hoxa10/Kv4.3 axis regulated myocardial hypertrophy and electrical remodeling caused by Ang II, thus providing new therapeutic strategies for myocardial hypertrophy and arrhythmias clinically, as well as opening up new fields and expanding new horizons for researchers.

## Data Availability

The raw data supporting the conclusions of this article will be made available by the authors, without undue reservation.
